# Outcomes of platelet-rich plasma for plantar fasciopathy: a best-evidence synthesis

**DOI:** 10.1186/s13018-020-01783-7

**Published:** 2020-09-21

**Authors:** Tao Yu, Jiang Xia, Bing Li, Haichao Zhou, Yunfeng Yang, Guangrong Yu

**Affiliations:** Department of Orthopedic Surgery, Tongji Hospital, Tongji University School of Medicine, Shanghai, 200065 China

**Keywords:** Platelet-rich plasma, Corticosteroid, Fasciopathy, Therapy

## Abstract

**Background:**

Plantar fasciopathy (PF) is a very common disease, affecting about 1/10 people in their lifetime. Platelet-rich plasma (PRP) had been demonstrated to be useful in achieving helpful effects for plantar fasciopathy. The purpose of this study was to compare the pain and functional outcomes between PRP and corticosteroid (CS) or placebo for plantar fasciopathy through meta-analysis and provide the best evidence.

**Methods:**

Literature was searched systematically to explore related studies that were published in Cochrane Library, PubMed, Embase, Medline, SpringerLink, OVID, and ClinicalTrials.gov. Articles regarding comparative research about the outcomes of PRP therapy and CS or placebo injection were selected. Data of pain and functional outcomes was extracted and imported into Reviewer Manager 5.3 to analyze.

**Results:**

Thirteen RCTs were included and analyzed. Analysis results showed significant superiority of PRP in outcome scores when compared with CS (VAS: MD = − 0.85, *P* < 0.0001, *I*^2^ = 85%; AOFAS: MD = 10.05, *P* < 0.0001, *I*^2^ = 85%), whereas there is no statistical difference in well-designed double-blind trials (VAS: MD = 0.15, *P* = 0.72, *I*^2^ = 1%; AOFAS: MD = 2.71, *P* = 0.17, *I*^2^ = 0%). In the comparison of the PRP and the placebo, the pooled mean difference was − 3.76 (*P* < 0.0001, 95% CI = − 4.34 to − 3.18).

**Conclusions:**

No superiority of PRP had been found in well-designed double-blind studies, whereas it is implied that the outcomes of PRP are better than placebo based on available evidence.

## Background

Plantar fasciitis (PF) is a very common disease, affecting about 1/10 people in their lifetime and subsequently affected the quality of life [1]. A variety of treatments had been carried out for plantar fasciopathy, including orthoses [2, 3], shockwave therapy [4, 5], drug medication [6], stretching exercise [7, 8], laser therapy [9], taping [10], and percutaneous injection [11]. But PF is difficult to cure completely. Platelet-rich plasma (PRP) is most concisely defined as a volume of plasma that contains a concentrate of platelets above that of baseline blood levels [12]. In recent years, the role of PRP in the treatment of PF has drawn wide attention [13–19]. However, the advantages of PRP in modern treatment for PF have not been fully confirmed, and different randomized controlled trials have drawn inconsistent conclusions when comparing the use of PRP with CS or placebo [20, 21]. The method of injecting corticosteroids (CS) is another common treatment that has proven to be effective [22, 23], but it has some limitations at the same time. Some studies [24, 25] have reported that different injection sites can produce pain of different lengths. On the other hand, this method may also lead to some complications, such as rupture of the plantar fascia and pad atrophy.

The systematic review of the validity of PRP and CS in the previous literature is inconsistent. Many literatures [26–29] indicate that the efficacy of PRP is significantly better than that of CS, but the reliability of these conclusions is affected to some extent by research selection strategies, statistical methods, and interference factors, such as limited number of trials. Thus, the aim of our systematic review was conducted to assess the efficacy of PRP for PF in terms of pain and functional outcomes. We performed the best comprehensive analysis of the evidence for the previous RCT. To ensure a more accurate conclusion as a means of guiding clinical decision-making, we combined and quantified PRP with CS or placebo for PF on clinical results.

## Material

### Literature search and study selection

Our systematic review of RCTs was produced to identify all of the published data on PRP used in PF according to the Cochrane Collaboration guidelines and best-evidence synthesis principles. The following processes are completed by two independent auditors. If no agreement can be reached through negotiation, another auditor will make the final decision. This study complies with the PRISMA guidelines, and all the information required was reported.

The databases retrieved include Cochrane Library, PubMed, Embase, Medline, SpringerLink, OVID, and ClinicalTrials.gov in December 2019. The following search terms were used: Plantar Fasciitis or Plantar Fasciopathy or PF, Platelet-Rich Plasma or PRP, Corticosteroid or CS, Randomized controlled trials or RCTs, and Intra-articular injection or IA injection.

The inclusion criteria of the studies are as follows: (1) must be RCT; (2) ensure at least 20 participants; (3) all participants are followed for at least 1 month; (4) use plantar fascia thickness (PFT) quantitative scores such as visual analog scale (VAS), tenderness threshold (TT), and heel tenderness index (HTI) which are used to assess pain, function, etc., as well as foot function index (FFI); (5) no more than 20% of participant loss occurred in follow-up; and (6) the article language must be English and the full text is available.

### Data extraction

The following data were extracted from included studies: study design, study population type, age, number of cases, and interventions. Any disagreements in data extraction were dealt with discussion and determined by a third researcher. Pain and function scores such as VAS, PFT, HTI, TT, and FFI were recorded at each visit. The mean and standard deviation (SD) was estimated with the following formula when it was not available in some included studies: SD = √*n* × (*P*_97.5_ − *P*_2.5_)/[2 × (= tin v(1 − 0.95, *n* − 1))]. *P*_97.5_ and *P*_2.5_ represented percentiles, and *n* was the number of the cases.

### Quality and risk of bias assessments

Two independent authors were responsible for reviewing all articles and rated the articles as “high,” “low,” or “unclear” based on the following: performance bias, detection bias, wear bias, reported bias, and other deviations. Any differences must be discussed for consensus. If no agreement can be reached, the third investigator must be consulted. A study of high quality should have appropriate distribution concealment, adopt a double-blind design, and report complete results data, and the experiment is considered to have a lower risk of bias [30, 31].

### Statistical analysis

RevMan 5.3.5 was used to analyze numerical data for included trials. For binary data, risk ratio (RR) and 95% confidence interval (CI) assessment test criteria were used (*ɑ* = 0.05). For continuous data, the SD in the meta-analysis was combined into a weighted mean difference (WMD) and a 95% CI. The heterogeneity was tested by *I*^2^ statistic. The heterogeneity was considered low when *I*^2^ statistic was 25–50%. The heterogeneity was moderate when *I*^2^ statistic was 50~75%, and the heterogeneity was high with *I*^2^ statistic > 75%. The sensitivity analysis was performed when the *I*^2^ statistic was > 50%. The difference was considered significant when the *P* value was < 0.05.

## Results

### Description of studies and demographic characteristics

**Fig. 1:**
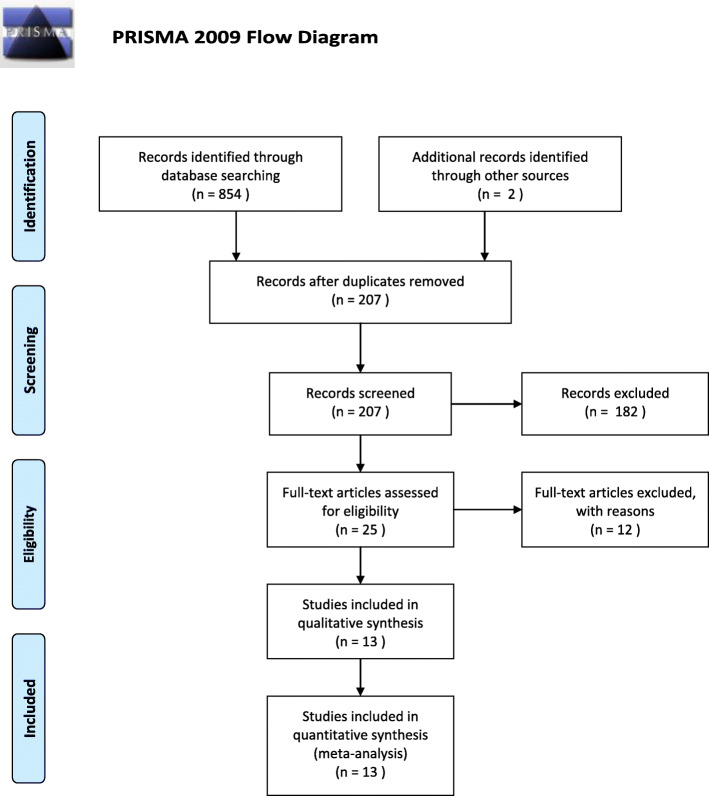
Flow chart outlining the process of study identification, inclusion, and exclusion

A total of 854 articles have been identified as potential correlation studies, as shown in Fig. 1. A total of 25 complete publications were screened after screening titles and abstracts (*n* = 182) and deleting copies (*n* = 649). Then, a comprehensive evaluation of 25 complete manuscripts was carried out, which further excluded the other 12 studies, and the remaining 13 studies were included. Of the 13 articles, 11 compared PRP and CS, with placebo (saline) as a control. Characteristics of the included studies are shown in Table 1. The classification of PRP was applied using the method described by Dohan Ehrenfest et al. [44].

**Table 1:** The characteristics of included studies

Study(Year)	Sex,n^**a**^,(M/F)^**b**^	Age,Mean(SD)	Groups,n		Treatment Schedules		Assessment Methods	Follow-up, Month
			PRP	CS/PL	PRP	CS/PL		
Omar AS (2012) [32]	**30(0/30)**	**PRP:42.5(17.5)** **CS:44.5(15.5)**	**15**	**15**	**1 dose of P-PRP was injected**	**1 dose of CS injected**	VAS; FHSQ	1
Lee TG (2007) [33]	**64/(4/57)**	**PRP:48.3(10.5)** **CS:49.2(11.1)**	**30**	**31**	**1 dose(1.5 ml) of autologous blood mixed with 1 ml of Lignocaine HCL 2% was injected**	**a combination of 20 mg (0.5 ml of a 40 mg/ml solution) of Triamcinolone Acetonide with 2 ml of Lignocaine HCL 1% was used**	VAS; TT	1;3;6
Aksahin E (2012) [34]	**60(25/35)**	**PRP:46.36(8.49)** **CS:45.47(9.36)**	**30**	**30**	**1 dose(3mL) P-PRP was injected after 2 mL of 2% prilocaine injection**	**1 does of 2 mL of 40 mg Methylprednisolone with 2 mL of 2% prilocaine was injected**	VAS; MRMS	6
Carlos AO (2017) [35]	**32(6/24)**	**PRP:44.8(0)** **CS:44.8(0)**	**15**	**15**	**1 dose of P-PRP(3 mL) activated with 0.45 mL of 10% calcium gluconate was injected**	**1 dose (8 mg) of dexamethasone plus 2 mL of lidocaine was injected**	VAS; FADI; AOFAS	1;2;3;4
Tiwari M (2013) [36]	**60(3/57)**	**NC**	**30**	**30**	**1 dose(5ml) of P-PRP was injected**	**1 dose of CS was injected**	VAS	1;3;6
Monto RR (2014) [13]	**40(17/23)**	**PRP:59(0)** **CS:51(0)**	**20**	**20**	**a single ultra-sound guided injection of autologous P-PRP was injected**	**a single ultrasound guided injection of 40 mg DepoMedrol cortisone**	AOFAS	3;6;12;24
Jain K (2015) [37]	**46(6/40)**	**PRP:55.6(0)** **CS:55.6(0)**	**23**	**23**	**1 dose(2.5ml) of P-PRP was injected**	**1 dose of Triamcinolone (Kenalog) 40 mg and Levobupivacaine hydrochloride (Chirocaine) injection**	VAS; AOFAS; MRMS	3;6;12
Say F (2014) [38]	**50(11/39)**	**PRP:47(0)** **CS:48.6(0)**	**25**	**25**	**1 dose(2.5ml) of P-PRP was injected**	**1 dose of a mixture of 40 mg/1 ml of methylprednisolone and 1 ml of prilocaine was injected**	VAS; AOFAS	6
Sherpy NA (2016) [39]	**50(13/37)**	**PRP:37.48(8.75)** **CS:38.52(6.2)**	**25**	**25**	**1 dose(3ml) of P-PRP was injected**	**1 dose of triamcinolone acetonide (2ml ,40 mg/ml) was injected**	VAS; FHSQ	1.5;3
Vahdatpour B (2016) [40]	**32(9/23)**	**PRP:45.44(7.74)** **CS:47.12(10.70)**	**16**	**16**	**1 dose(3ml) of P-PRP was injected**	**1 dose of Methylprednisolone( 1 ml) plus lidocaine( 1 ml) was injected**	VAS; MRMS	1;3;6
Jain SK (2018) [41]	**80(46/34)**	**PRP:37.7(10.3)** **CS:38.9(9.5)**	**40**	**40**	**1 dose(3ml) of P-PRP was injected**	**1 dose of methyl prednisolone (2m,40 mg) with 2% lidocaine hydrochloride(2 mL) was injected**	VAS; MRMS; AAOS; FAI; AOFAS	1;3;6
Sarah JL (2019) [42]	**28(9/19)**	**PRP:47.9(10.702)** **Placebo:52.1(10.277)**	**14**	**14**	**1 dose(3ml) of P-PRP was injected**	**1 dose of saline injected**	VAS	6
Mahindra P (2016) [43]	**75(31/44)**	**PRP:30.72(7.42)** **CS:33.92(8.61)** **Pl:35.48(9.54)**	**25**	**25**	**1 dose(3ml) of P-PRP was injected**	**CS:1 dose of CS injected** **PL:1 dose of saline injected**	VAS; AOFAS	3

*NC* Not clear, *M* Male, *F* Female, *PL* Placebo *VAS* visual analog scale, *FHSQ* foot health status questionnaire, *TT* tenderness threshold, *MRMS* ModiWed Roles and Maudsley score, *FADI* Foot Ankle Disability Index, *AOFAS* American Orthopaedic Foot and Ankle Society, *FAI* Foot and Ankle Outcome Instrument, *AAOS* merican Academy of Orthopedic Surgeons

Methodological quality evaluation revealed that seven trials [13, 32, 33, 35, 36, 38, 40] had a low risk of bias, and the other six trials had a high or moderate risk. The risk of bias of included studies is demonstrated in Table 2.

**Table 2:** The methodological quality of included RCTs

Author(Year)	A	B	C	D	E	F	G	H	Overall Quality
PRP Versus CS									
Omar AS (2012) [32]	√	×	×	×	×	×	〇	〇	Low
Lee TG (2007) [33]	〇	×	×	×	×	×	〇	√	Low
Aksahin E (2012) [34]	√	〇	√	√	√	×	√	√	High
Carlos AO (2017) [35]	√	×	×	〇	×	×	〇	√	Low
Tiwari M (2013) [36]	√	×	×	×	×	×	〇	〇	Low
Monto RR (2014) [13]	√	×	×	×	〇	×	〇	√	Low
Jain K (2015) [37]	√	×	〇	×	√	〇	√	√	Moderate
Say F (2014) [38]	√	×	×	×	×	×	√	〇	Low
Sherpy NA (2016) [39]	√	〇	×	×	√	√	√	〇	Moderate
Vahdatpour B (2016) [40]	√	×	〇	×	×	×	〇	〇	Low
Jain SK (2018) [41]	√	√	√	〇	√	√	×	√	High
PRP Versus PL									
Sarah JL (2019) [42]	√	〇	×	×	√	√	√	〇	Moderate
Mahindra P (2016) [43]	√	〇	√	√	×	√	√	√	High

A, adequate sequence generation; B, allocation concealment; C, blinding (participants); D, blinding (investigators); E, blinding (evaluators); F, incomplete outcome data inexistent or addressed; G, free of selective reporting; H, free of other bias

High, well-designed double-blind trials with proper allocation concealment and complete outcome data; Moderate: double-blind trials without proper allocation concealment or complete outcome data or single-blind trials; Low: trials without proper blinding methods applied.

The check mark (√), yes; cross mark (×), no; and circle (〇), unclear

### PRP versus CS

**Fig. 2:**
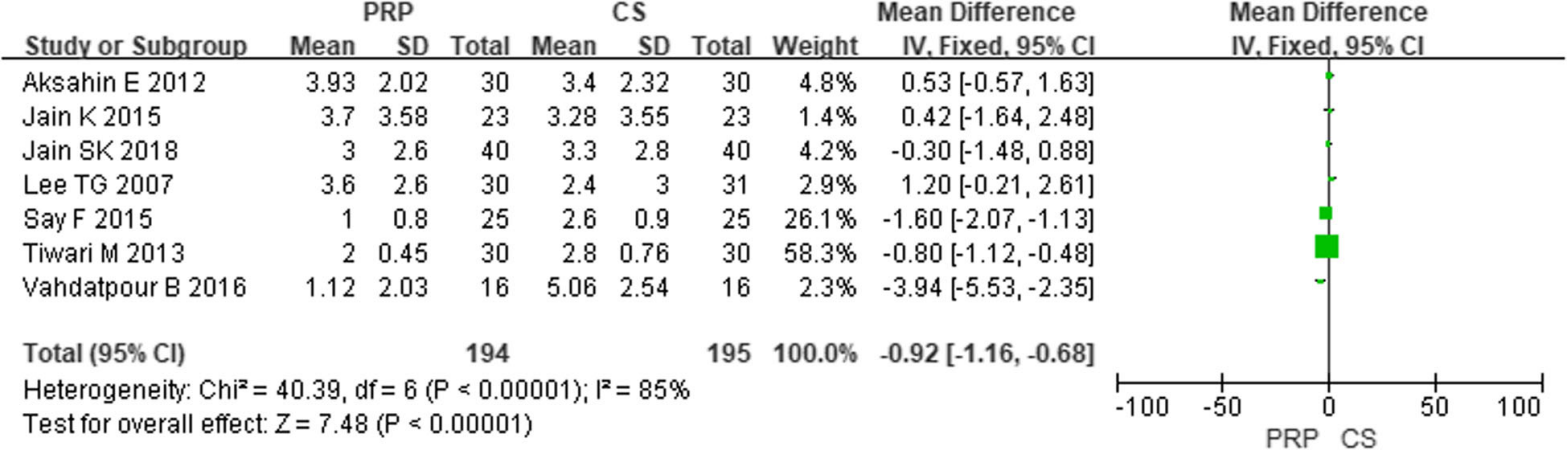
Forest plot of VAS in the PRP group compared with the CS group from the 6-month follow-up

**Fig. 3:**
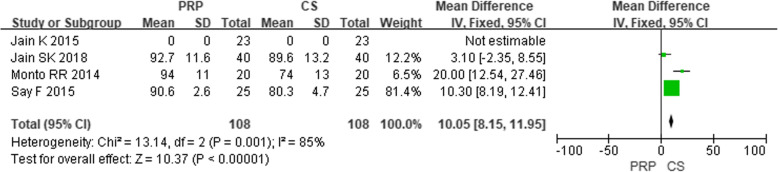
Forest plot of AOFAS in the PRP group compared with the CS group from the 6-month follow-up

**Fig. 4:**
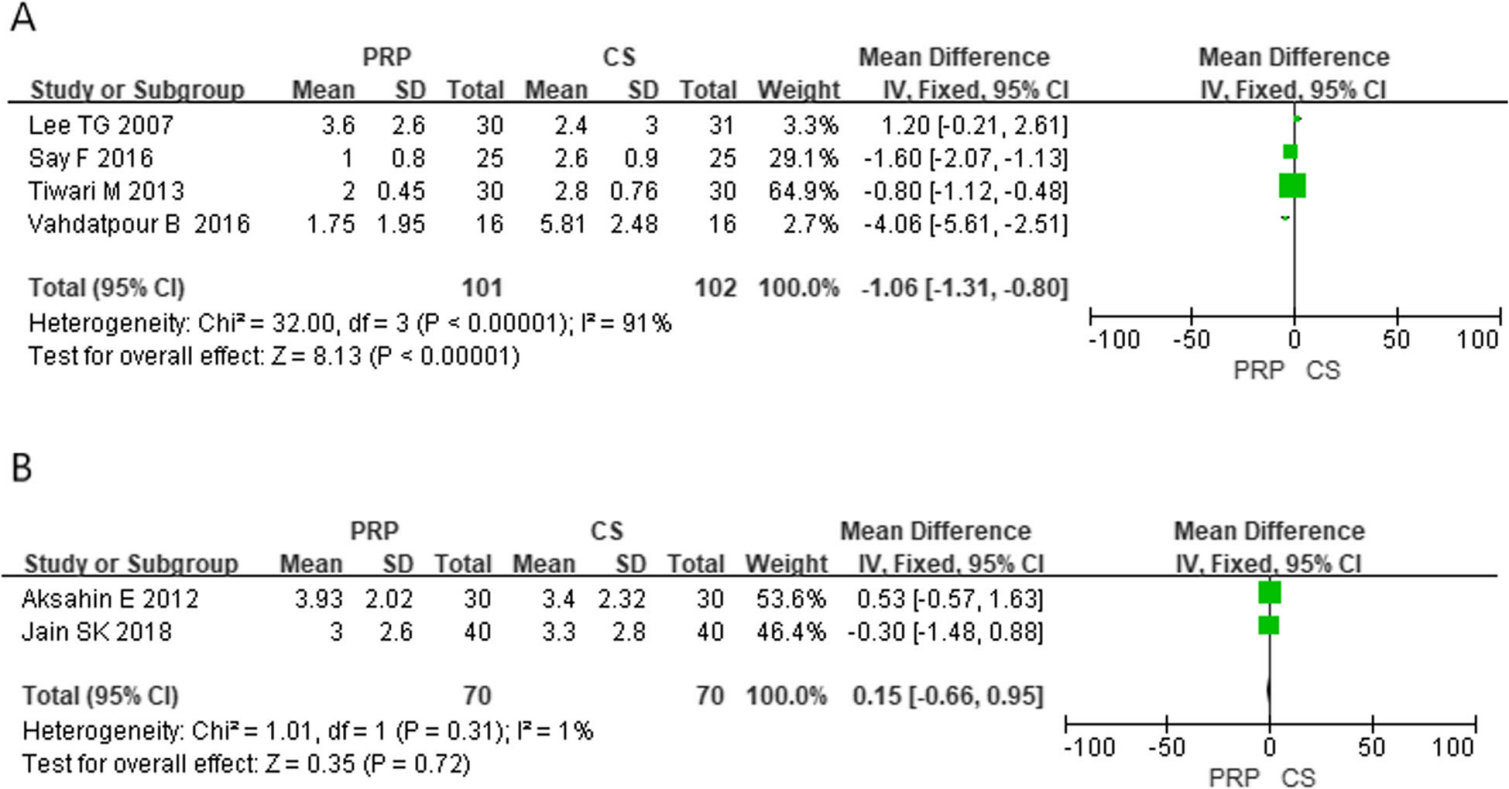
Forest plot of subgroup analysis of VAS of the different levels in methodology

**Fig. 5:**
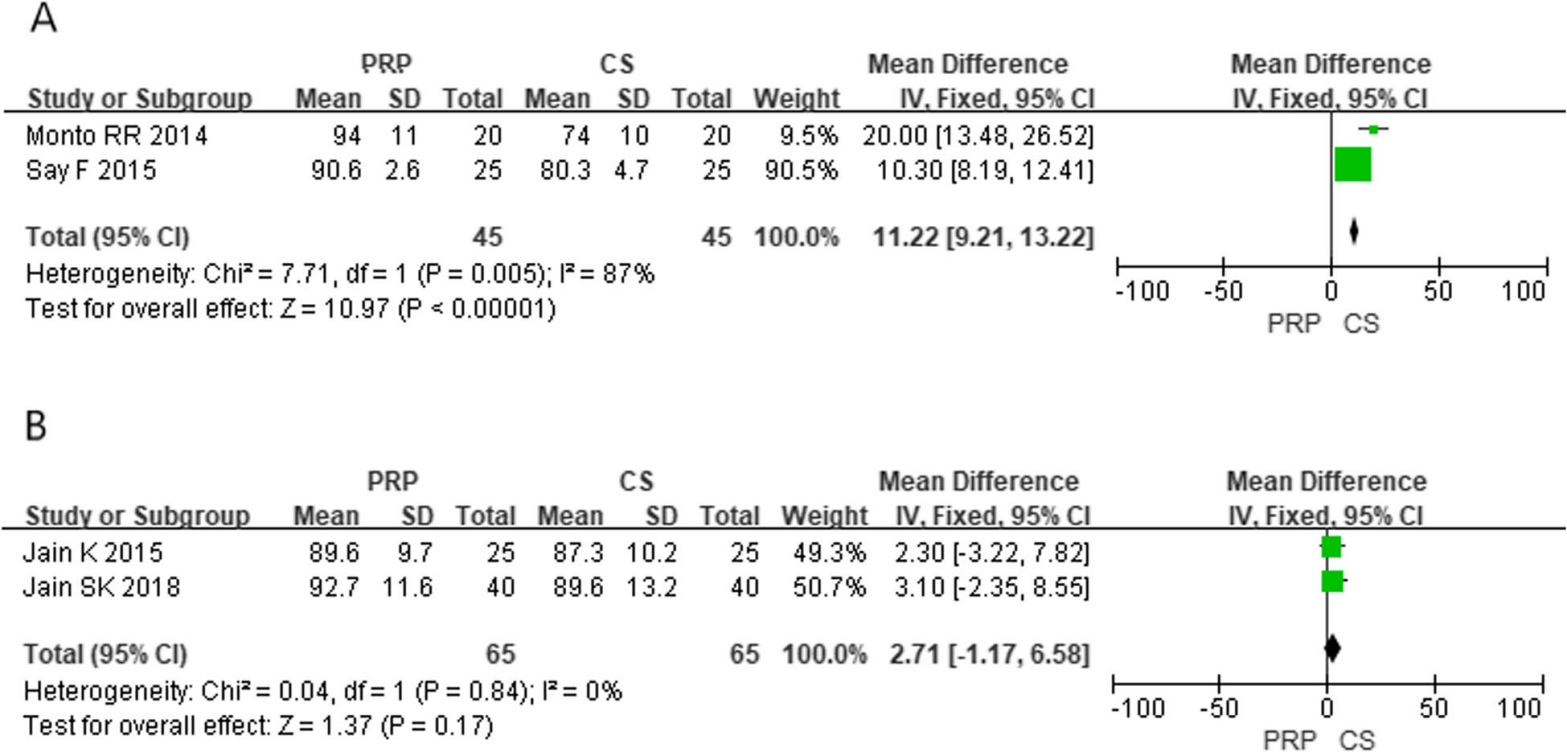
Forest plot of subgroup analysis of AOFAS of the different levels in methodology

In these 11 studies of the comparative efficacy of PRP and CS, 7 studies provided VAS scores for 6 months after injection and 4 studies provided an AOFAS score for 6 months after injection. After 6 months of follow-up, the combined effect of VAS and AOFAS was calculated and PRP was found to be superior to CS by calculating the pooled effect size of VAS and AOFAS respectively at the follow-up of 6 months (Fig. 2, MD = − 0.92, *P* < 0.00001, *I*^2^ = 85%; Fig. 3, MD = 10.05, *P* < 0.00001, *I*^2^ = 85%). The sensitivity analysis failed to identify any trial that might lead to such statistical heterogeneity. We further analyzed the subgroup at different levels of VAS, as shown in Fig. 4 (low quality: MD = − 1.06, *P* < 0.0001, *I*^2^ = 91%; high quality: MD = 0.15, *P* = 0.72, *I*^2^ = 1%). Further, a subgroup analysis of different levels of AOFAS was performed, as shown in Fig. 5 (low quality: MD = 11.22, *P* < 0.0001, *I*^2^ = 87%; moderate and high quality: MD = 2.71, *P* = 0.17, *I*^2^ = 0%). No significant heterogeneity was observed in both high-quality and moderate-quality trials. For longer or shorter follow-up periods, most moderate- and/or high-quality trials had reported VAS and AOFAS scores within 3 and/or 12 months. After calculating the combined effect size, we found that PRP had no significant advantage during the follow-up period of 3 months and 12 months (VAS—3 months: MD = − 1.10, *P* = 0.21, *I*^2^ = 92%; 12 months: MD = − 8.73, *P* = 0.36, *I*^2^ = 97%; AOFAS—3 months: MD = 3.10, *P* = 0.17, *I*^2^ = 92%; 12 months: MD = 8.23, *P* = 0.06, *I*^2^ = 96%).

### PRP versus PL

**Fig. 6:**
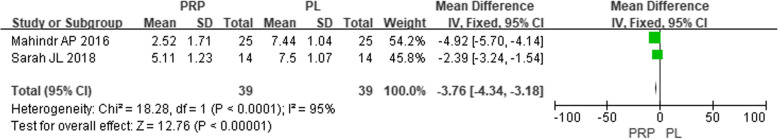
Forest plot of VAS in the PRP group compared with the PL group

Two studies comparing PRP and placebo were included in this meta-analysis [42, 43]. One article was judged to have an ambiguous bias risk; the other was found to be at a lower risk (Table 2). The pooled effect size of the VAS was calculated and is shown in Fig. 6 (MD = − 3.76, *P* < 0.00001, *I*^2^ = 95%). Sensitivity analysis found no cause for heterogeneity. In addition, only one trial [43] using AOFAS as measurement had obviously revealed the beneficial effects of PRP.

## Discussion

In this system review, we made a comprehensive evaluation of the efficacy of PRP comparing with CS or PL. By considering the therapeutic effects of all trials, we think that PRP is more effective than CS, but when only moderate- and/or high-quality literature was included for analysis, PRP had no obvious advantage. However, significant differences were found in the direct comparison between PRP and placebo. Thus, based on the best evidence, although PRP is not superior to CS in terms of pain relief and functional improvement, it is still considered more effective than placebo. With the application of best-evidence synthesis, the results of this study are not consistent with the conclusions of most previous meta-analysis [26–28]. In this study, we integrated all of the moderate- and/or high-quality randomized controlled trials on the efficacy of PRP in the treatment of PF and further explained more clearly the significance of the comparison of different interventions.

The good blind design of the study can play a key role in the assessment of the results of different interventions. Aksahin et al. [34] performed three blind experiments due to the difficulty in obtaining a different intra-group injection experience for blind operators. The studies of Jain et al. [41] and Mahindra et al. [43] that had relatively large sample size and used the blinding strategy for both participants and researchers were considered to be high-quality studies. Jain et al. [37] and Sherpy et al. [39] performed trials of different time in the patient, but the participants were not allowed to remain blind. Another experiment conducted by Johnson-Lynn et al. [42] performed a double-blind design, but its selective reporting and allocation of uncertainty concealment reduced the quality of the method. Because blind methods were not applied, the other trials were considered high risk of bias. Since the meta-analysis of the two high-quality tests did not produce the beneficial effect of PRP on CS and the homogeneity was high, it can be concluded that PRP may not be more effective than CS in relieving pain and improving function.

We all know that statistical heterogeneity can be reduced by accurate blind design, but heterogeneity will still exist in the final methodology, which is indeed a difficult problem. In different tests, the production of PRP and CS, as well as the dosage, time, and interval, was not completely consistent. In addition, differences in population, outcome scores, and disease duration may lead to high heterogeneity and different clinical outcomes. All these inconsistencies increase the difficulty of data synthesis and may lead to incorrect conclusions.

Just like other studies, our research also has some limitations. First of all, the number of included experiments with lower bias risk is relatively small, and because of the interference of external factors, it is impossible for literature searchers to retrieve all the experiments that may meet the requirements. Secondly, the current review focuses only on articles published in English. Inclusion in other languages may have an impact on heterogeneity and change current outcomes. In addition, the follow-up time of different studies was not consistent. In order to better verify the efficacy of PRP, a more large sample size of the strict randomized controlled trial needs to be further designed. Last but not least, limited by the reported results in included studies, this study only evaluated the VAS and AOFAS. Some other indicators were not analyzed. For instance, ultrasound is not only a diagnostic method but also a useful tool for monitoring response to treatments in patients with plantar fasciitis [45, 46]. It can be included in the analysis in the future study when the relevant literature is sufficient.

## Conclusions

In conclusion, in a well-designed double-blind trial, PRP did not show a better curative effect than CS, and in most of the researches, the advantage of PRP may be due to the lack of blind method. However, according to current evidence, PRP is still considered to be more effective than placebo.

## Data Availability

All data generated or analyzed during this study are included in this published article.

## Abbreviations

PRP: Platelet-rich plasma
CS: Corticosteroid
PL: Placebo
NC: Not clear
M: Male
F: Female
VAS: Visual analog scale
FHSQ: Foot health status questionnaire
TT: Tenderness threshold
MRMS: ModiWed Roles and Maudsley score
FADI: Foot Ankle Disability Index
AOFAS: American Orthopaedic Foot and Ankle Society
FAI: Foot and Ankle Outcome Instrument
AAOS: American Academy of Orthopedic Surgeons
